# 1-[4-(2,3,4,6-Tetra-*O*-acetyl-β-d-allo­pyranos­yloxy)benzyl­idene]thio­semi­carbazide

**DOI:** 10.1107/S1600536809007260

**Published:** 2009-03-06

**Authors:** Li Fu, Xiu-juan Yin, Lei Zheng, Ying Li, Shu-fan Yin

**Affiliations:** a College of Chemistry, Sichuan University, Chengdu 610064, People’s Republic of China

## Abstract

The title compound, C_22_H_27_N_3_O_10_S, was synthesized by reaction of an ethanol solution of helicid (systematic name: 4-formylphenl-β-d-allopyranoside), thio­semicarbazide and acetic acid. The mol­ecule exhibits a *trans* conformation with respect to the C=N double bond. The pyran ring adopts a chair conformation. In the crystal structure, the mol­ecules are linked into chains parallel to the *b* axis by inter­molecular N—H⋯O hydrogen bonds.

## Related literature

For the synthesis and biological activity of helicid, see: Chen *et al.* (1981 ▶); Sha & Mao (1987 ▶); Zhu *et al.* (2006 ▶); Yang *et al.* (2008 ▶); Wen *et al.* (2007 ▶).
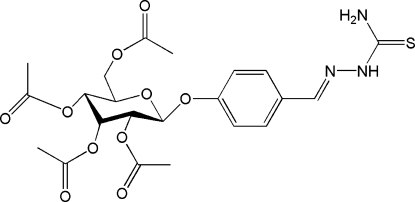


## Experimental

### 

#### Crystal data

C_22_H_27_N_3_O_10_S
*M* *_r_* = 525.53
Orthorhombic, 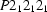

*a* = 9.848 (3) Å
*b* = 11.515 (3) Å
*c* = 23.250 (4) Å
*V* = 2636.5 (12) Å^3^
*Z* = 4
Mo *K*α radiation
μ = 0.18 mm^−1^
*T* = 292 K
0.54 × 0.46 × 0.24 mm

#### Data collection

Enraf-Nonius CAD-4 diffractometer
Absorption correction: ψ scan (North *et al.*, 1968 ▶) *T*_min_ = 0.909, *T*_max_ = 0.958
5423 measured reflections
4940 independent reflections
2851 reflections with *I* > 2σ(*I*)
*R*_int_ = 0.008
3 standard reflections every 200 reflections intensity decay: 1.0%

#### Refinement

*R*[*F* ^2^ > 2σ(*F* ^2^)] = 0.062
*wR*(*F* ^2^) = 0.195
*S* = 1.07
4940 reflections
329 parameters
H atoms treated by a mixture of independent and constrained refinement
Δρ_max_ = 0.30 e Å^−3^
Δρ_min_ = −0.27 e Å^−3^
Absolute structure: Flack, (1983 ▶), 2108 Friedel pairs
Flack parameter: 0.35 (19)

### 

Data collection: *DIFRAC* (Gabe *et al*., 1993 ▶); cell refinement: *DIFRAC*; data reduction: *NRCVAX* (Gabe *et al.*, 1989 ▶); program(s) used to solve structure: *SHELXS97* (Sheldrick, 2008 ▶); program(s) used to refine structure: *SHELXL97* (Sheldrick, 2008 ▶); molecular graphics: *ORTEP-3 for Windows* (Farrugia, 1997 ▶); software used to prepare material for publication: *SHELXL97*.

## Supplementary Material

Crystal structure: contains datablocks global, I. DOI: 10.1107/S1600536809007260/rz2297sup1.cif

Structure factors: contains datablocks I. DOI: 10.1107/S1600536809007260/rz2297Isup2.hkl

Additional supplementary materials:  crystallographic information; 3D view; checkCIF report

## Figures and Tables

**Table 1:** Hydrogen-bond geometry (Å, °)

*D*—H⋯*A*	*D*—H	H⋯*A*	*D*⋯*A*	*D*—H⋯*A*
N2—H2*N*1⋯O10^i^	0.75 (5)	2.33 (5)	3.076 (6)	172 (6)
N3—H3*A*⋯O8^ii^	0.86	2.60	3.229 (7)	131

Symmetry codes: (i) 
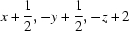
; (ii) 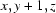.

**Fig. 1.**
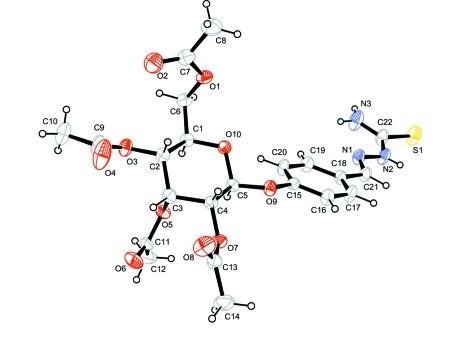
The molecular structure of the title compound, with displacement ellipsoids drawn at the 30% probability level.

## supplementary crystallographic information

### Comment

The natural compound helicid (systematic name: 4-formylphenl-β-D-allopyranoside), which is extracted from the fruit of *Helicia nilagirica* Beed. (Chen *et al.*, 1981), has been one major active ingredient of herb medicine used in China for a long time. It has manifested good biological effects on the central nervous system and low toxicity (Sha & Mao, 1987). Some derivatives of this compound have been reported to possess good pharmacological activity (Zhu *et al.*, 2006; Yang *et al.*, 2008). We report here the crystal structure of the title compound, whose synthesis has been already reported elsewhere (Wen *et al.*, 2007).

In the molecule of the title compound (Fig. 1), the pyran ring adopts a chair conformation, with the hydroxy group at C3 in axial position and the other substituents at C1, C2 and C4 in equatorial positions. The average C–C bond length within the pyran ring is 1.524 (3) Å. The molecule exhibits a *trans* conformation with respect to the N1═C21 double bond, as indicated by the value of the C21–N1–N2–C22 torsion angle of -171.8 (6)°. In the crystal packing, intermolecular N—H···O hydrogen bonds (Table 1) link the molecules into chains running parallel to the *b* axis.

### Experimental

A mixture of helicid (0.45 g, 1 mmol), acetic acid (0.5 ml) and thiosemicarbazide (0.09 g, 1 mmol) in ethanol (15 ml) was refluxed for 3 h. After cooling to room temperature, the precipitate was filtered, washed with ether and recrystallized from 95% alcohol to give a white powder. Colourless crystals suitable for X-ray analysis were obtained by slow evaporation of an ethanol/dichloromethane (4:1 v/v) solution at room temperature.

### Refinement

The H atom bound to N2 was located in a difference Fourier map and refined freely. All other H atoms were positioned geometrically and refined using a riding model, with C—H = 0.93–0.97 Å, N—H = 0.86 Å, and *U*_iso_(H) = 1.2*U*_eq_ (C, N) or 1.5*U*_eq_ (C) for methyl H atoms.

### Crystal data

**Table:**

C_22_H_27_N_3_O_10_S	*F*(000) = 1104
*M**_r_* = 525.53	*D*_x_ = 1.324 Mg m^−^^3^
Orthorhombic, *P*2_1_2_1_2_1_	Mo *K*α radiation, λ = 0.71073 Å
Hall symbol: P 2ac 2ab	Cell parameters from 19 reflections
*a* = 9.848 (3) Å	θ = 4.5–7.5°
*b* = 11.515 (3) Å	µ = 0.18 mm^−^^1^
*c* = 23.250 (4) Å	*T* = 292 K
*V* = 2636.5 (12) Å^3^	Block, colourless
*Z* = 4	0.54 × 0.46 × 0.24 mm

### Data collection

**Table:**

Enraf-Nonius CAD-4 diffractometer	2851 reflections with *I* > 2σ(*I*)
Radiation source: fine-focus sealed tube	*R*_int_ = 0.008
graphite	θ_max_ = 26.0°, θ_min_ = 1.8°
ω/2–θ scans	*h* = −10→12
Absorption correction: ψ scan (North *et al.*, 1968)	*k* = −14→14
*T*_min_ = 0.909, *T*_max_ = 0.958	*l* = −28→28
5423 measured reflections	3 standard reflections every 200 reflections
4940 independent reflections	intensity decay: 1.0%

### Refinement

**Table:**

Refinement on *F*^2^	Secondary atom site location: difference Fourier map
Least-squares matrix: full	Hydrogen site location: mixed
*R*[*F*^2^ > 2σ(*F*^2^)] = 0.062	H atoms treated by a mixture of independent and constrained refinement
*wR*(*F*^2^) = 0.195	*w* = 1/[σ^2^(*F*_o_^2^) + (0.1108*P*)^2^] where *P* = (*F*_o_^2^ + 2*F*_c_^2^)/3
*S* = 1.07	(Δ/σ)_max_ = 0.001
4940 reflections	Δρ_max_ = 0.30 e Å^−^^3^
329 parameters	Δρ_min_ = −0.27 e Å^−^^3^
0 restraints	Absolute structure: Flack, (1983), 2108 Friedel pairs
Primary atom site location: structure-invariant direct methods	Flack parameter: 0.35 (19)

### Special details

**Table:**

Geometry. All e.s.d.'s (except the e.s.d. in the dihedral angle between two l.s. planes) are estimated using the full covariance matrix. The cell e.s.d.'s are taken into account individually in the estimation of e.s.d.'s in distances, angles and torsion angles; correlations between e.s.d.'s in cell parameters are only used when they are defined by crystal symmetry. An approximate (isotropic) treatment of cell e.s.d.'s is used for estimating e.s.d.'s involving l.s. planes.
Refinement. Refinement of *F*^2^ against ALL reflections. The weighted *R*-factor *wR* and goodness of fit *S* are based on *F*^2^, conventional *R*-factors *R* are based on *F*, with *F* set to zero for negative *F*^2^. The threshold expression of *F*^2^ > σ(*F*^2^) is used only for calculating *R*-factors(gt) *etc*. and is not relevant to the choice of reflections for refinement. *R*-factors based on *F*^2^ are statistically about twice as large as those based on *F*, and *R*- factors based on ALL data will be even larger.

### Fractional atomic coordinates and isotropic or equivalent isotropic displacement parameters (Å^2^)

**Table:**

	*x*	*y*	*z*	*U*_iso_*/*U*_eq_	
S1	0.67386 (19)	0.79792 (13)	1.07682 (8)	0.0785 (5)	
O1	0.0580 (3)	0.0411 (3)	0.84568 (15)	0.0535 (9)	
O2	−0.0599 (4)	−0.0865 (4)	0.7938 (2)	0.0862 (14)	
O3	0.2305 (3)	−0.1128 (3)	0.71708 (14)	0.0522 (9)	
O4	0.1797 (6)	−0.3014 (4)	0.7185 (2)	0.1061 (18)	
O5	0.5080 (3)	−0.0914 (3)	0.74617 (14)	0.0482 (8)	
O6	0.6199 (5)	−0.2413 (4)	0.70896 (19)	0.0865 (14)	
O7	0.6077 (3)	−0.1962 (3)	0.84617 (15)	0.0501 (8)	
O8	0.5379 (4)	−0.3793 (3)	0.8569 (2)	0.0781 (13)	
O9	0.4971 (3)	−0.0280 (3)	0.92000 (13)	0.0443 (7)	
O10	0.3267 (3)	0.0069 (3)	0.85614 (12)	0.0407 (7)	
N1	0.6208 (4)	0.4935 (4)	1.00575 (18)	0.0548 (11)	
N2	0.6680 (5)	0.5844 (4)	1.0393 (2)	0.0565 (12)	
H2N1	0.708 (5)	0.569 (5)	1.066 (2)	0.049 (17)*	
N3	0.5385 (6)	0.7104 (5)	0.9885 (2)	0.0832 (17)	
H3A	0.5134	0.6535	0.9670	0.100*	
H3B	0.5078	0.7791	0.9820	0.100*	
C1	0.2764 (4)	0.0066 (4)	0.7979 (2)	0.0402 (10)	
H1	0.3406	0.0512	0.7745	0.048*	
C2	0.2779 (4)	−0.1174 (4)	0.7760 (2)	0.0433 (11)	
H2	0.2159	−0.1649	0.7990	0.052*	
C3	0.4199 (5)	−0.1666 (4)	0.7796 (2)	0.0459 (12)	
H3	0.4230	−0.2467	0.7654	0.055*	
C4	0.4693 (4)	−0.1589 (4)	0.8422 (2)	0.0396 (10)	
H4	0.4119	−0.2072	0.8669	0.048*	
C5	0.4627 (4)	−0.0333 (4)	0.86160 (19)	0.0367 (10)	
H5	0.5245	0.0149	0.8387	0.044*	
C6	0.1424 (4)	0.0717 (5)	0.7977 (2)	0.0496 (13)	
H6A	0.1602	0.1546	0.7987	0.060*	
H6B	0.0943	0.0548	0.7623	0.060*	
C7	−0.0460 (5)	−0.0308 (5)	0.8376 (3)	0.0597 (14)	
C8	−0.1389 (6)	−0.0288 (6)	0.8892 (3)	0.0787 (19)	
H8A	−0.1704	−0.1061	0.8971	0.118*	
H8B	−0.2152	0.0206	0.8814	0.118*	
H8C	−0.0903	0.0003	0.9220	0.118*	
C9	0.1755 (6)	−0.2085 (6)	0.6944 (3)	0.0694 (16)	
C10	0.1184 (7)	−0.1847 (6)	0.6369 (3)	0.083 (2)	
H10A	0.0842	−0.2555	0.6206	0.125*	
H10B	0.1880	−0.1535	0.6125	0.125*	
H10C	0.0457	−0.1295	0.6403	0.125*	
C11	0.6074 (5)	−0.1380 (5)	0.7148 (2)	0.0541 (14)	
C12	0.6992 (6)	−0.0490 (6)	0.6898 (3)	0.0775 (19)	
H12A	0.7702	−0.0867	0.6686	0.116*	
H12B	0.7382	−0.0034	0.7202	0.116*	
H12C	0.6484	0.0006	0.6645	0.116*	
C13	0.6297 (6)	−0.3110 (5)	0.8532 (2)	0.0576 (14)	
C14	0.7786 (6)	−0.3377 (6)	0.8568 (3)	0.084 (2)	
H14A	0.8104	−0.3227	0.8951	0.126*	
H14B	0.8272	−0.2896	0.8301	0.126*	
H14C	0.7934	−0.4179	0.8475	0.126*	
C15	0.5342 (4)	0.0805 (4)	0.9425 (2)	0.0401 (10)	
C16	0.6049 (5)	0.0769 (4)	0.9935 (2)	0.0512 (12)	
H16	0.6249	0.0062	1.0109	0.061*	
C17	0.6458 (5)	0.1805 (5)	1.0185 (2)	0.0565 (14)	
H17	0.6946	0.1788	1.0527	0.068*	
C18	0.6155 (5)	0.2860 (4)	0.9935 (2)	0.0439 (11)	
C19	0.5441 (5)	0.2877 (4)	0.9419 (2)	0.0516 (13)	
H19	0.5235	0.3583	0.9245	0.062*	
C20	0.5032 (5)	0.1840 (4)	0.9160 (2)	0.0483 (12)	
H20	0.4558	0.1848	0.8814	0.058*	
C21	0.6587 (5)	0.3937 (4)	1.0228 (2)	0.0500 (12)	
H21	0.7155	0.3884	1.0546	0.060*	
C22	0.6230 (6)	0.6925 (5)	1.0308 (2)	0.0585 (14)	

### Atomic displacement parameters (Å^2^)

**Table:**

	*U*^11^	*U*^22^	*U*^33^	*U*^12^	*U*^13^	*U*^23^
S1	0.1123 (13)	0.0390 (8)	0.0844 (11)	0.0062 (8)	−0.0385 (10)	−0.0059 (8)
O1	0.0362 (16)	0.058 (2)	0.067 (2)	−0.0041 (15)	0.0009 (15)	−0.0004 (19)
O2	0.077 (3)	0.083 (3)	0.098 (4)	−0.023 (2)	−0.002 (2)	−0.018 (3)
O3	0.062 (2)	0.044 (2)	0.050 (2)	−0.0105 (17)	−0.0127 (16)	−0.0017 (16)
O4	0.170 (5)	0.046 (3)	0.103 (4)	−0.036 (3)	−0.061 (3)	0.005 (3)
O5	0.0532 (18)	0.0387 (18)	0.0527 (19)	0.0070 (16)	0.0063 (15)	−0.0047 (15)
O6	0.096 (3)	0.075 (3)	0.088 (3)	0.035 (3)	0.004 (3)	−0.031 (3)
O7	0.0487 (17)	0.0349 (18)	0.067 (2)	0.0089 (15)	−0.0076 (17)	−0.0047 (16)
O8	0.082 (3)	0.031 (2)	0.122 (4)	−0.003 (2)	−0.022 (2)	−0.005 (2)
O9	0.0551 (17)	0.0317 (16)	0.0461 (18)	−0.0059 (15)	−0.0085 (15)	−0.0055 (15)
O10	0.0422 (15)	0.0364 (17)	0.0435 (18)	0.0015 (14)	−0.0017 (14)	−0.0034 (14)
N1	0.062 (3)	0.049 (3)	0.053 (3)	−0.002 (2)	−0.016 (2)	−0.009 (2)
N2	0.077 (3)	0.035 (2)	0.058 (3)	0.003 (2)	−0.029 (3)	0.000 (2)
N3	0.120 (5)	0.046 (3)	0.084 (4)	0.004 (3)	−0.046 (3)	0.003 (3)
C1	0.045 (2)	0.024 (2)	0.051 (3)	−0.0048 (19)	0.000 (2)	0.000 (2)
C2	0.050 (3)	0.030 (2)	0.050 (3)	−0.003 (2)	−0.004 (2)	0.000 (2)
C3	0.056 (3)	0.028 (2)	0.054 (3)	0.000 (2)	−0.003 (2)	−0.008 (2)
C4	0.042 (2)	0.024 (2)	0.052 (3)	0.0039 (18)	−0.005 (2)	−0.007 (2)
C5	0.044 (2)	0.023 (2)	0.043 (3)	0.0005 (19)	0.0009 (19)	−0.0030 (19)
C6	0.043 (3)	0.050 (3)	0.056 (3)	0.001 (2)	0.000 (2)	0.008 (3)
C7	0.053 (3)	0.045 (3)	0.081 (4)	−0.002 (3)	−0.005 (3)	−0.001 (3)
C8	0.058 (3)	0.084 (5)	0.095 (4)	−0.019 (3)	0.014 (3)	0.004 (4)
C9	0.083 (4)	0.055 (4)	0.070 (4)	−0.020 (3)	−0.028 (3)	−0.010 (3)
C10	0.099 (5)	0.080 (5)	0.071 (4)	−0.022 (4)	−0.034 (4)	−0.001 (3)
C11	0.060 (3)	0.058 (4)	0.045 (3)	0.028 (3)	−0.006 (3)	−0.018 (3)
C12	0.062 (3)	0.096 (5)	0.075 (4)	0.023 (3)	0.020 (3)	0.005 (4)
C13	0.076 (4)	0.039 (3)	0.058 (3)	0.024 (3)	−0.018 (3)	−0.012 (3)
C14	0.070 (4)	0.066 (4)	0.117 (6)	0.025 (3)	−0.019 (4)	−0.028 (4)
C15	0.041 (2)	0.032 (2)	0.048 (3)	0.004 (2)	−0.003 (2)	−0.007 (2)
C16	0.069 (3)	0.032 (3)	0.052 (3)	0.002 (2)	−0.013 (3)	0.003 (2)
C17	0.069 (3)	0.053 (3)	0.048 (3)	−0.002 (3)	−0.023 (2)	0.002 (3)
C18	0.054 (3)	0.030 (2)	0.048 (3)	−0.001 (2)	−0.005 (2)	−0.010 (2)
C19	0.062 (3)	0.035 (3)	0.057 (3)	0.000 (2)	−0.013 (2)	−0.004 (2)
C20	0.062 (3)	0.035 (3)	0.048 (3)	−0.008 (2)	−0.018 (2)	−0.003 (2)
C21	0.060 (3)	0.040 (3)	0.050 (3)	0.003 (2)	−0.015 (2)	−0.009 (2)
C22	0.076 (3)	0.047 (3)	0.053 (3)	−0.004 (3)	−0.016 (3)	0.007 (3)

### Geometric parameters (Å, °)

**Table:**

S1—C22	1.693 (6)	C4—H4	0.9800
O1—C7	1.330 (6)	C5—H5	0.9800
O1—C6	1.435 (6)	C6—H6A	0.9700
O2—C7	1.210 (7)	C6—H6B	0.9700
O3—C9	1.336 (6)	C7—C8	1.510 (8)
O3—C2	1.448 (6)	C8—H8A	0.9600
O4—C9	1.207 (7)	C8—H8B	0.9600
O5—C11	1.333 (6)	C8—H8C	0.9600
O5—C3	1.452 (6)	C9—C10	1.477 (8)
O6—C11	1.204 (7)	C10—H10A	0.9600
O7—C13	1.349 (6)	C10—H10B	0.9600
O7—C4	1.432 (6)	C10—H10C	0.9600
O8—C13	1.201 (7)	C11—C12	1.485 (8)
O9—C5	1.401 (5)	C12—H12A	0.9600
O9—C15	1.404 (5)	C12—H12B	0.9600
O10—C5	1.422 (5)	C12—H12C	0.9600
O10—C1	1.442 (5)	C13—C14	1.501 (8)
N1—C21	1.271 (6)	C14—H14A	0.9600
N1—N2	1.386 (6)	C14—H14B	0.9600
N2—C22	1.336 (7)	C14—H14C	0.9600
N2—H2N1	0.75 (5)	C15—C16	1.374 (7)
N3—C22	1.307 (7)	C15—C20	1.376 (7)
N3—H3A	0.8600	C16—C17	1.387 (7)
N3—H3B	0.8600	C16—H16	0.9300
C1—C2	1.516 (6)	C17—C18	1.380 (7)
C1—C6	1.518 (6)	C17—H17	0.9300
C1—H1	0.9800	C18—C19	1.391 (7)
C2—C3	1.512 (7)	C18—C21	1.477 (7)
C2—H2	0.9800	C19—C20	1.397 (7)
C3—C4	1.536 (6)	C19—H19	0.9300
C3—H3	0.9800	C20—H20	0.9300
C4—C5	1.517 (6)	C21—H21	0.9300
			
C7—O1—C6	119.3 (4)	C7—C8—H8C	109.5
C9—O3—C2	118.3 (4)	H8A—C8—H8C	109.5
C11—O5—C3	119.4 (4)	H8B—C8—H8C	109.5
C13—O7—C4	117.1 (4)	O4—C9—O3	122.3 (5)
C5—O9—C15	117.6 (3)	O4—C9—C10	126.7 (5)
C5—O10—C1	114.0 (3)	O3—C9—C10	111.0 (5)
C21—N1—N2	114.1 (4)	C9—C10—H10A	109.5
C22—N2—N1	120.7 (5)	C9—C10—H10B	109.5
C22—N2—H2N1	121 (4)	H10A—C10—H10B	109.5
N1—N2—H2N1	117 (4)	C9—C10—H10C	109.5
C22—N3—H3A	120.0	H10A—C10—H10C	109.5
C22—N3—H3B	120.0	H10B—C10—H10C	109.5
H3A—N3—H3B	120.0	O6—C11—O5	122.3 (6)
O10—C1—C2	108.3 (4)	O6—C11—C12	125.2 (5)
O10—C1—C6	107.5 (4)	O5—C11—C12	112.5 (5)
C2—C1—C6	118.2 (4)	C11—C12—H12A	109.5
O10—C1—H1	107.5	C11—C12—H12B	109.5
C2—C1—H1	107.5	H12A—C12—H12B	109.5
C6—C1—H1	107.5	C11—C12—H12C	109.5
O3—C2—C3	111.4 (4)	H12A—C12—H12C	109.5
O3—C2—C1	106.3 (4)	H12B—C12—H12C	109.5
C3—C2—C1	110.1 (4)	O8—C13—O7	122.0 (5)
O3—C2—H2	109.7	O8—C13—C14	126.6 (5)
C3—C2—H2	109.7	O7—C13—C14	111.4 (5)
C1—C2—H2	109.7	C13—C14—H14A	109.5
O5—C3—C2	107.4 (4)	C13—C14—H14B	109.5
O5—C3—C4	106.5 (4)	H14A—C14—H14B	109.5
C2—C3—C4	108.9 (4)	C13—C14—H14C	109.5
O5—C3—H3	111.3	H14A—C14—H14C	109.5
C2—C3—H3	111.3	H14B—C14—H14C	109.5
C4—C3—H3	111.3	C16—C15—C20	121.7 (4)
O7—C4—C5	107.9 (3)	C16—C15—O9	115.3 (4)
O7—C4—C3	110.2 (4)	C20—C15—O9	123.0 (4)
C5—C4—C3	108.9 (4)	C15—C16—C17	118.8 (5)
O7—C4—H4	109.9	C15—C16—H16	120.6
C5—C4—H4	109.9	C17—C16—H16	120.6
C3—C4—H4	109.9	C18—C17—C16	121.2 (4)
O9—C5—O10	107.5 (3)	C18—C17—H17	119.4
O9—C5—C4	108.6 (3)	C16—C17—H17	119.4
O10—C5—C4	108.9 (3)	C17—C18—C19	119.0 (4)
O9—C5—H5	110.6	C17—C18—C21	118.9 (4)
O10—C5—H5	110.6	C19—C18—C21	122.1 (4)
C4—C5—H5	110.6	C18—C19—C20	120.4 (5)
O1—C6—C1	112.3 (4)	C18—C19—H19	119.8
O1—C6—H6A	109.1	C20—C19—H19	119.8
C1—C6—H6A	109.1	C15—C20—C19	118.8 (4)
O1—C6—H6B	109.1	C15—C20—H20	120.6
C1—C6—H6B	109.1	C19—C20—H20	120.6
H6A—C6—H6B	107.9	N1—C21—C18	122.0 (4)
O2—C7—O1	122.4 (5)	N1—C21—H21	119.0
O2—C7—C8	127.4 (5)	C18—C21—H21	119.0
O1—C7—C8	110.2 (5)	N3—C22—N2	118.0 (5)
C7—C8—H8A	109.5	N3—C22—S1	123.5 (5)
C7—C8—H8B	109.5	N2—C22—S1	118.5 (4)
H8A—C8—H8B	109.5		
			
C21—N1—N2—C22	−171.8 (6)	C7—O1—C6—C1	−102.7 (5)
C5—O10—C1—C2	61.6 (4)	O10—C1—C6—O1	−43.1 (5)
C5—O10—C1—C6	−169.7 (3)	C2—C1—C6—O1	79.8 (5)
C9—O3—C2—C3	84.1 (5)	C6—O1—C7—O2	12.4 (8)
C9—O3—C2—C1	−156.0 (4)	C6—O1—C7—C8	−166.7 (4)
O10—C1—C2—O3	−178.4 (3)	C2—O3—C9—O4	−9.7 (9)
C6—C1—C2—O3	59.1 (5)	C2—O3—C9—C10	173.3 (5)
O10—C1—C2—C3	−57.6 (5)	C3—O5—C11—O6	6.7 (7)
C6—C1—C2—C3	179.9 (4)	C3—O5—C11—C12	−172.2 (4)
C11—O5—C3—C2	−141.7 (4)	C4—O7—C13—O8	−1.1 (7)
C11—O5—C3—C4	101.7 (5)	C4—O7—C13—C14	−180.0 (5)
O3—C2—C3—O5	60.0 (5)	C5—O9—C15—C16	−161.2 (4)
C1—C2—C3—O5	−57.7 (5)	C5—O9—C15—C20	18.6 (6)
O3—C2—C3—C4	174.9 (4)	C20—C15—C16—C17	−0.1 (8)
C1—C2—C3—C4	57.2 (5)	O9—C15—C16—C17	179.8 (5)
C13—O7—C4—C5	154.5 (4)	C15—C16—C17—C18	0.7 (8)
C13—O7—C4—C3	−86.7 (5)	C16—C17—C18—C19	−0.9 (8)
O5—C3—C4—O7	−59.8 (4)	C16—C17—C18—C21	178.5 (5)
C2—C3—C4—O7	−175.3 (4)	C17—C18—C19—C20	0.3 (8)
O5—C3—C4—C5	58.4 (4)	C21—C18—C19—C20	−179.0 (5)
C2—C3—C4—C5	−57.2 (5)	C16—C15—C20—C19	−0.4 (8)
C15—O9—C5—O10	−79.8 (4)	O9—C15—C20—C19	179.7 (5)
C15—O9—C5—C4	162.5 (4)	C18—C19—C20—C15	0.3 (8)
C1—O10—C5—O9	180.0 (3)	N2—N1—C21—C18	178.3 (5)
C1—O10—C5—C4	−62.5 (4)	C17—C18—C21—N1	−171.7 (5)
O7—C4—C5—O9	−65.2 (4)	C19—C18—C21—N1	7.6 (8)
C3—C4—C5—O9	175.2 (4)	N1—N2—C22—N3	−3.1 (9)
O7—C4—C5—O10	178.1 (3)	N1—N2—C22—S1	174.7 (4)
C3—C4—C5—O10	58.5 (4)		

### Hydrogen-bond geometry (Å, °)

**Table:**

*D*—H···*A*	*D*—H	H···*A*	*D*···*A*	*D*—H···*A*
N2—H2N1···O10^i^	0.75 (5)	2.33 (5)	3.076 (6)	172 (6)
N3—H3A···O8^ii^	0.86	2.60	3.229 (7)	131

Symmetry codes: (i) *x*+1/2, −*y*+1/2, −*z*+2; (ii) *x*, *y*+1, *z*.
